# Developing the Link-me+EMPHN Mental Health Model of Care to Improve General Practitioner Capacity for Mental Health Care in Australian Primary Care: Protocol for a Mixed Methods Formative Study

**DOI:** 10.2196/79560

**Published:** 2026-01-23

**Authors:** Amy Coe, Jane London, Anne-Marie Martin, Aaron van Ree, Kirsty Lembke, Caroline Johnson, Bridget Bassilios, Catherine Kaylor-Hughes

**Affiliations:** 1 Department of General Practice and Primary Care The University of Melbourne Parkville, Victoria Australia; 2 Eastern Melbourne Primary Health Network Box Hill, Victoria Australia; 3 Melbourne School of Population and Global Health The University of Melbourne Carlton, Victoria Australia

**Keywords:** mental health, decision support, model of care, implementation, primary care, general practice, co-design, formative, Theoretical Domains Framework

## Abstract

**Background:**

The rising prevalence of mental health conditions continues to place significant pressure on general practitioners (GPs) and general practice. Despite their critical role in managing mental health conditions, GPs and practice staff face substantial barriers in providing effective mental health care, such as time and financial constraints. A new mental health model of care (Link-me+EMPHN) aimed at engaging and building capacity in GPs and practice staff for the provision of mental health services is being designed and implemented in a Primary Health region in Victoria, Australia.

**Objective:**

This protocol describes formative research that aims to support the implementation of the Link-me+EMPHN Mental Health Model of Care in general practice, focusing on identifying current gaps in GP mental health training, assessing barriers and facilitators to implementation, exploring stakeholder perceptions and experiences, and determining needs and priorities for successful integration into routine practice.

**Methods:**

The formative research involves a multimethod approach comprising (1) a desktop audit of currently available mental health training and guidelines for GPs; (2) up to 20 semistructured interviews with GPs and practice nurses; (3) an online survey exploring current mental health care practice of GPs and practice nurses; (4) two co-design workshops with people with lived experience of mental health care help-seeking in general practice and four expert working group workshops with a multidisciplinary primary-care team; and (5) testing of the model of care in a simulated practice setting with GPs, patients, and Care Navigators. The desktop audit, online survey, and interviews will be mapped to the Theoretical Domains Framework to systematically identify gaps in GP knowledge and skills in providing mental health care. Thematic analysis of the interviews will provide context for the gaps found in current mental health care and training so that we can begin to address these. Findings from each of the co-design, expert working group, and simulation sessions will be thematically analyzed and will include key themes, insights, and any practical implications.

**Results:**

The formative research received funding in May 2024, with the formative research components taking place from May 2024 to July 2025. As of June 24, 2025, a total of 5 people participated in co-design, 14 in semistructured interviews, 28 in simulation sessions, 30 completed the online survey, and 4 working groups have been held. The desktop audit yielded 270 results. The findings of the formative research are tentatively planned for publication in 2026.

**Conclusions:**

This formative research will generate a robust understanding of the factors influencing GP and practice engagement in mental health care and will provide practical solutions to improve implementation of the mental health model of care. The findings will contribute to creating a sustainable and scalable model that improves mental health outcomes for patients and supports GPs.

**International Registered Report Identifier (IRRID):**

DERR1-10.2196/79560

## Introduction

### Background

Mental disorders are a significant global health concern, affecting approximately 14% of the population worldwide within a given year, while lifetime prevalence estimates exceed 30%-50% depending on the study and region [1,2]. In Australia, the burden is also significant, with 45% of people experiencing a mental health condition at some point in their lives and 1 in 5 adults facing a mental disorder each year [3]. Anxiety and affective disorders are the most common, affecting 17% and 8% of Australians each year, respectively [3]. Mental and physical health problems are frequently comorbid, with individuals who experience mental health conditions often having higher rates of chronic physical illnesses such as heart disease, diabetes, and obesity, leading to more complex care needs and worse health outcomes overall [4,5]. Additionally, as neurodevelopmental disorders like attention-deficit/hyperactivity disorder (ADHD) are becoming increasingly recognized in children, adolescents, and adults, there is an increasing need to incorporate ADHD care within the broader scope of mental health care [6,7]. While ADHD is primarily classified as a neurodevelopmental disorder, it is closely linked to mental health, frequently co-occurring with conditions such as anxiety, depression, and substance use disorders, and significantly impacting an individual’s mental health and well-being [8,9]. Despite growing awareness of mental ill-health, delivering mental health care remains a challenge, especially in primary care settings. General practitioners (GPs), who are often the first point of professional contact for individuals seeking help with their mental health, play a pivotal role in diagnosing and managing these conditions [10].

In 2021, it was estimated that 38% of Australian general practice consultations involve a mental health component; however, it is thought that this number is greatly underreported [11]. Mental health conditions often require long-term management, complex care coordination, and a nuanced understanding of both psychological and physical health, placing GPs under increasing pressure to balance mental health care with other clinical duties and patient needs [12]. Eighty-three percent of Australian GPs report spending significant unpaid time coordinating care and follow-up for patients with mental health conditions, putting further strain on an already time-poor setting [11]. Over 70% of Australian GPs also agree that mental health consultations take longer than general consultations, creating logistical problems in their practice with an equal number of GPs reporting that mental health work is tiring and emotionally draining [11]. The cost of providing these longer, more complex mental health consultations and coordinating care can also be a significant financial barrier for GPs, especially in rural and remote areas with limited resources [11,13]. GPs also frequently face systemic barriers that limit their ability to provide comprehensive mental health care including difficulties in accessing multidisciplinary teams, such as psychologists and psychiatrists [14], which are crucial for complex mental health cases. This fragmentation between primary care and mental health services can result in delays and gaps in patient care and a lack of continuity in care [15,16]. Even when GPs are well-equipped with theoretical and experiential knowledge, the lack of real-time advice and guidance in managing complex mental health cases, disconnected care pathways, and inadequate infrastructure can prevent them from applying these skills in a holistic manner [15-18]. Together, these factors contribute to reluctance among GPs to take on more mental health patients, unless they have a particular interest in this field [11,19].

### Implementing Collaborative Care

The challenges of managing mental health in primary care have led to the development of collaborative care models. Originating in the 1990s with the work of Wayne Katon and colleagues, these models were developed to address the significant gap between the prevalence of mental health conditions in primary care and the capacity of GPs to effectively manage them [20]. The core principles of collaborative care include team-based care delivery, population-based care, measurement-based treatment to target severity, and evidence-based treatment [21,22]. Over the past 30 years, numerous studies have demonstrated the effectiveness of collaborative care in improving outcomes for patients with common mental health conditions such as depression and anxiety [23,24]. The first comprehensive Cochrane review and meta-analysis in 2012 showed that collaborative care was associated with significant improvement in depression and anxiety outcomes compared to usual care [22].

However, despite strong evidence supporting its effectiveness, the implementation of collaborative care in routine practice has faced persistent challenges [25-27]. In the United Kingdom, the Improving Access to Psychological Therapies (IAPT) program (also known as NHS Talking Therapies) has expanded into primary care to make psychological therapies more accessible by offering services in various community settings and through different modalities (face-to-face, telephone, and online) using a collaborative care model [28,29]. The implementation of the IAPT has faced ongoing challenges, including issues in identifying suitable patients, providing holistic care, and ensuring continuity of support [30]. A qualitative study also found that while a new Health and Wellbeing pathway within IAPT was perceived as having a positive impact on mental health, service- and individual-level barriers need to be addressed to enhance statutory and community support links, manage service users’ expectations, and improve accessibility for certain groups [31]. A Norwegian model showed promising results in terms of improving GPs’ ability to detect mental disorders in young people and potentially reducing long-term work disability through an adapted collaborative care model based on the successful Hamilton Family Health Team approach from Canada [32]. The model involved colocating psychologists and psychiatrists within general practice settings and demonstrated the feasibility of implementing core elements of collaborative care within a different health care context. However, it also highlighted significant challenges in sustaining such models without dedicated funding for collaborative activities, thus demonstrating the importance of aligning financial incentives with policy goals to support integrated care [32].

Australian Primary Health Networks (PHNs) are agencies that aim to strengthen the primary health care system in Australia. In their local catchment areas, PHNs (previously known as Medicare Locals and Divisions of General Practice) have been commissioning stepped primary mental health care since 2016 and commissioning and/or providing primary mental health care since 2001 [33-35]. Unlike IAPT, which serves the entire UK mental health help-seeking population, primary mental health care commissioned by PHNs targets disadvantaged groups, while universal primary mental health care has been available since 2006 via Australia’s *Better Access to Psychiatrists, Psychologists and GPs* under the Medicare Benefits Schedule [36]. PHNs are best placed to support the implementation of collaborative care into clinical practice. Working with PHNs for funding and implementation of collaborative care models offers several advantages: (1) PHNs have a deep understanding of local health needs and resources, allowing for tailored implementation; (2) PHNs can potentially pool resources from various funding streams to support collaborative care initiatives; and (3) PHNs are well-positioned to facilitate cross-sector collaboration, crucial for the success of integrated care models. By working with PHNs, successful collaborative care models may be more easily scaled up across different practices within a region [37,38]. PHNs can also play a key role in monitoring implementation and outcomes, providing valuable data for ongoing improvement and policy development. Additionally, they can support the training and development of primary care staff to enhance their skills in mental health care.

### GP Training

Alongside collaborative and stepped care models, emphasis has been placed on the provision of training to improve GP confidence and engagement in mental health care [39-41]. Various types of mental health training programs are available to GPs, ranging from short courses to more comprehensive programs [42]. These have been shown to increase GPs’ mental health diagnosis rates, improve their ability to provide counseling and stress management, and manage somatic symptoms, as well as increase GP confidence, motivation, attitudes, and skills in treating patients with mental ill-health [43-45]. In Australia, GPs are not required to complete additional mental health training after completing specialist training; however, a higher level of mental health training correlates with a higher level of billing that a GP may claim for some specific mental health consultations [42,46]. In 2020, the Australian Productivity Commission Inquiry into Mental Health released a report that found “most GPs receive minimal training in mental healthcare” [47]. This finding appears to contradict the fact that 90% of Australian GPs have completed the recommended entry-level mental health skills training required to be eligible for higher billing rebates for mental health treatment plans [11] and suggests that training may be insufficient to fully prepare GPs for the complexities of mental health care in clinical practice. GPs also report feeling inadequately supported to provide effective mental health care [16,41,44]. It has been suggested that this may be due to limited access to, or knowledge of, mental health training and resources, and a lack of infrastructure to support ongoing learning [48]. However, qualitative studies of GP barriers to providing mental health care suggest that there may also be a gap in training related to how to apply theoretical knowledge to real-life challenges when delivering mental health care in practice [12,16].

### The Link-me+EMPHN Mental Health Model of Care

**Figure 1:**
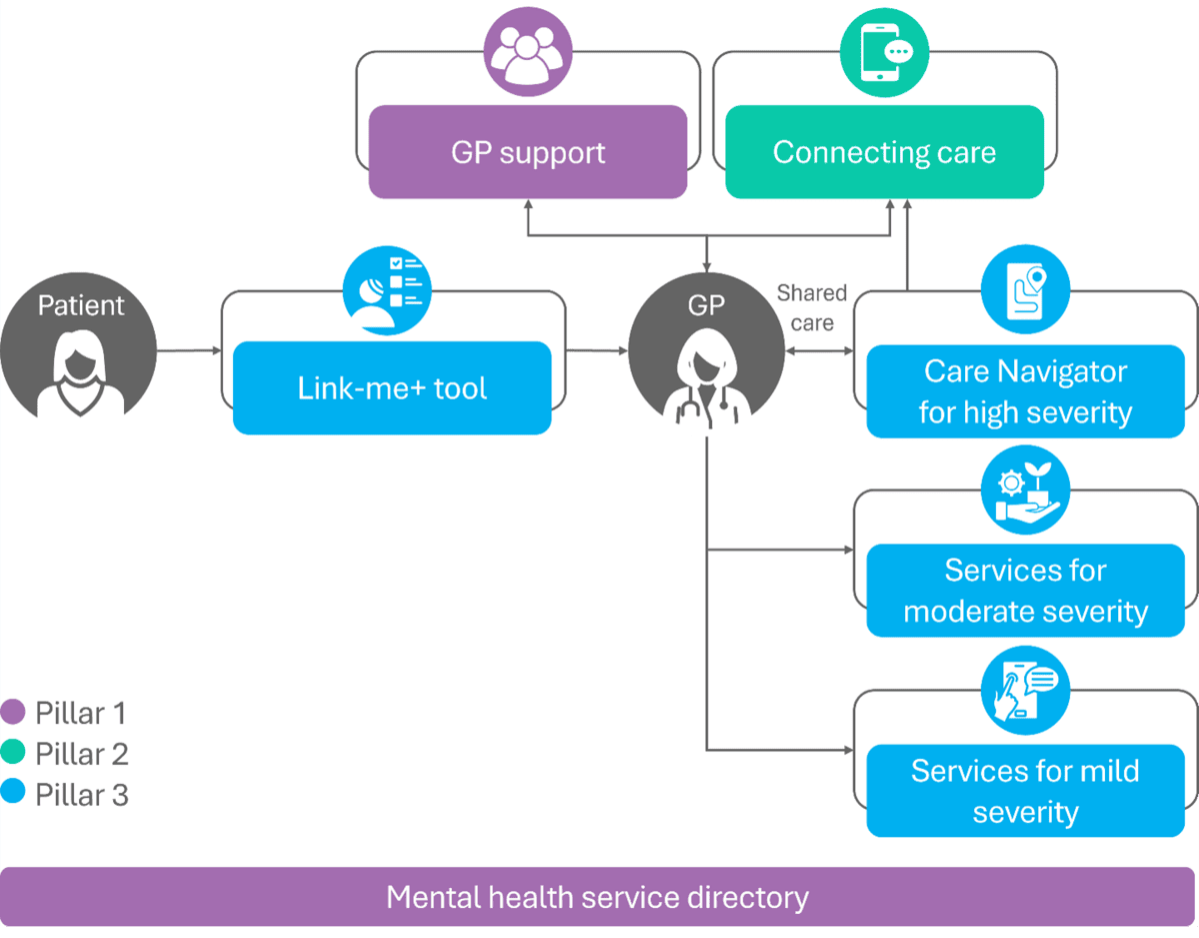
Expected flow of the 3 pillars of the Link-me+EMPHN Mental Health Model of Care. The Link-me+ tool (pillar 3) has been previously validated. Although patients initially use the tool independently, results are integrated into GP consultations. For detailed information on patient flow and GP-patient interactions when using Link-me+, please refer to Fletcher et al [49,50]. EMPHN: Eastern Melbourne Primary Health Network; GP: general practitioner.

To build capacity and support sustainable behavior change among GPs to provide best practice mental health care, the “Link-me+EMPHN Mental Health Model of Care” is being designed by the Eastern Melbourne Primary Health Network (EMPHN) and Primary Care Mental Health Research Team, University of Melbourne, in Victoria, Australia. The Link-me+EMPHN Mental Health Model of Care will consist of three pillars (Figure 1) [49,50].

#### Pillar 1: GP Support

EMPHN will work with subcatchment groups of general practices aiming to fill gaps in clinical practice and enhance skills and confidence in managing mental health. A comprehensive service directory for use across all 3 pillars by all relevant staff and patients will be created. This directory will aim to offer a range of features to enhance service accessibility and coordination.

#### Pillar 2: SupportConnect

It aims to enhance SupportConnect to support GPs with clinical and practical queries. SupportConnect is a website and telephone service staffed by mental health professionals that helps people to navigate the mental health system and find appropriate services [51]. It assists individuals, families, caregivers, and health professionals with mental health support, suicide prevention, and substance use issues. Current usage of SupportConnect by GPs is low. Between December 2024 and June 2025, only 12% of contacts made to SupportConnect were from GPs. Care Navigators (briefly described in pillar 3) will also use SupportConnect. SupportConnect has been renamed “Medicare Mental Health” as of November 2025.

#### Pillar 3: Link-me+

The existing Link-me+ model of care will be scaled for implementation into practices to assist GPs with triaging. Link-me+ includes a digitally supported, prognostic tool that uses a stepped care approach to stratify people with mental health issues and then matches treatment to their predicted symptom severity (in 3 months) and level of need [49,50,52]. As part of the Link-me+ model, a Care Navigator will be allocated to patients with severe mental health symptoms. The Care Navigator is a pivotal role in which a trained health professional collaborates with patients and their GPs to develop and implement a holistic, person-centered mental health care plan that is based on patient needs and priorities for recovery. Care navigation is a short-term intervention that aims to identify and link patients with appropriate services [49,50,53]. The Link-me+ prognostic tool has been tested in 2 randomized controlled trials (RCTs) and has been demonstrated to reduce psychological distress in patients with anxiety and depression, especially in those with severe symptoms [49,50]. Additionally, the Link-me+ model of care leads to better outcomes when patients follow the recommended treatment [50]. The prognostic tool was developed from data collected as part of the Diamond 10-year longitudinal study of 789 Victorian (Australia) general practice patients experiencing depressive symptoms [52]. User-centered design assisted the development of an interface to ensure the usability and acceptability within general practice settings for patients [54]. The Link-me+EMPHN Mental Health Model of Care, while primarily focused on depression and anxiety, can indirectly benefit patients with other mental health conditions due to the high rates of comorbidity [55-57] and the holistic, person-centered approach of the model. Therefore, the expert working group (including representatives from EMPHN and participating GPs) decided to incorporate ADHD as a focus in this project, alongside depression and social anxiety. This decision reflects the increasing recognition of adult ADHD in primary care settings and the need for improved management strategies. This project represents the first implementation of Link-me+ in routine general practice, transitioning it from a research tool to clinical use.

The Link-me+EMPHN Mental Health Model of Care will represent an innovative approach to stepped mental health care in general practice. The addition of pillars 1 and 2 to the existing Link-me+ model of care (pillar 3) represents a significant expansion of the original model, addressing identified needs in primary care mental health management and creating a more integrated, multifaceted approach to care delivery. This evolution from a single-focus intervention to a 3-pillar model may address the complex challenges of implementing collaborative mental health care in primary practice settings. To ensure its effectiveness and sustainability in routine practice, further formative research is necessary. Formative research is essential for developing effective interventions, programs, or strategies as it provides a deep understanding of the target population’s needs, behaviors, and context and ensures that the final product is tailored to the end user [58,59]. In this way, formative research supports the creation of evidence-based solutions that are more likely to be accepted and adopted by the target population, ultimately leading to more sustainable and impactful results [59,60]. The Link-me+EMPHN Mental Health Model of Care formative research detailed in this protocol aims to:

1. Identify typical mental health care and knowledge gaps in current GP mental health training and support.
2. Determine the barriers to and enablers of the implementation of the Link-me+EMPHN Mental Health Model of Care in routine practice by GPs and other practice staff.
3. Explore the perceptions, experiences, and other influencing factors for the acceptance and feasibility of the Link-me+EMPHN Mental Health Model of Care in practice.
4. Identify stakeholder needs and priorities for the implementation of the Link-me+EMPHN Mental Health Model of Care in practice.

The findings from this formative research will not only inform the overall implementation strategy for the Link-me+EMPHN Mental Health Model of Care but will also guide the specific design and framing of each element within the 3 pillars. This approach will ensure that each component within the 3 pillars is tailored to address the unique challenges and opportunities identified in the EMPHN region.

## Methods

### Overview

**Figure 2:**
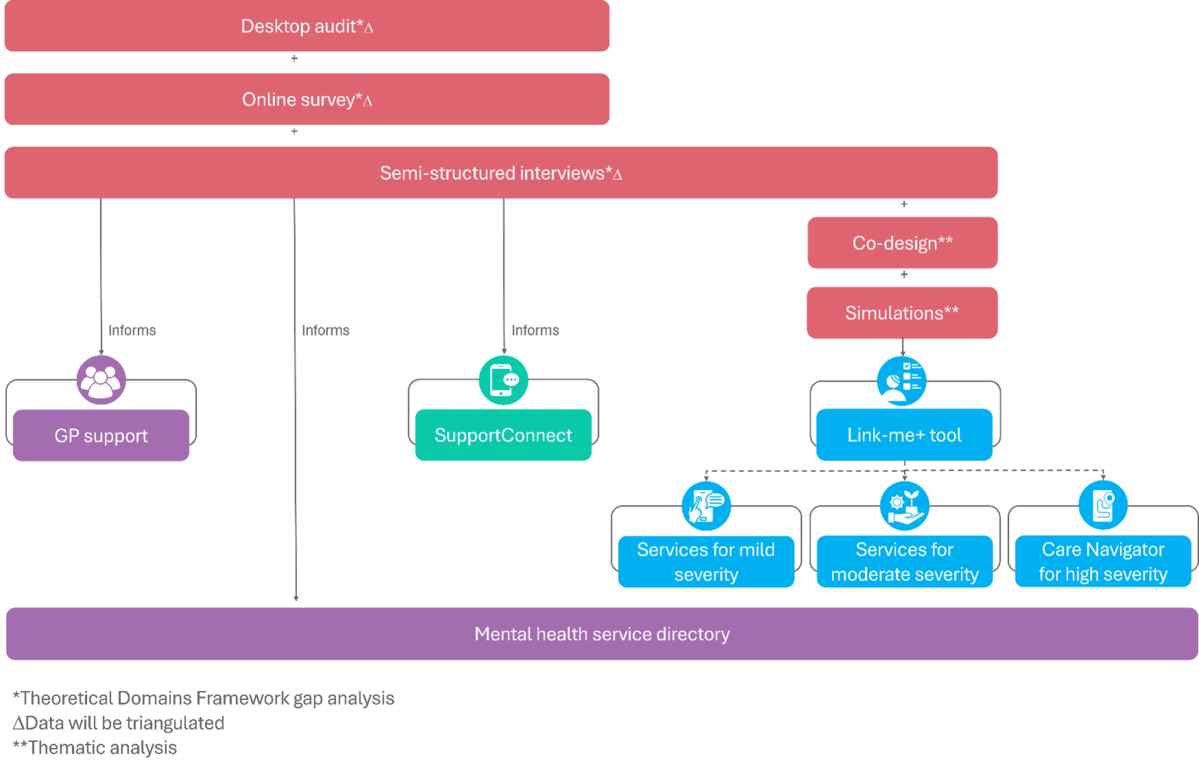
The multipart formative research design to inform the 3 pillars of the Link-me+EMPHN Mental Health Model of Care. EMPHN: Eastern Melbourne Primary Health Network; GP: general practitioner.

The formative research will consist of a multipart, mixed methods formative study to develop and implement the Link-me+EMPHN Mental Health Model of Care (Figure 2):

- Part 1: A desktop audit of currently available resources for GPs.
- Part 2: An online survey of GPs, practice nurses, practice managers, and service managers.
- Part 3: Semistructured interviews with GPs.
- Part 4: Co-design sessions with general practice patients with lived experience of mental health care.
- Part 5: GP clinic simulation.

Central to the formative research, the identification of knowledge gaps, implementation factors, and priorities will be mapped to the Theoretical Domains Framework (TDF) [61,62] to help develop tailored supports for the successful implementation of the Link-me+EMPHN Mental Health Model of Care. The TDF is a framework that provides a comprehensive way to analyze behaviors and identify factors influencing change [61,62]. It consists of 14 domains of behavior change, such as knowledge, skills, social/professional role and identity, beliefs about capabilities, and environmental context, allowing for the identification of cognitive, affective, social, and environmental factors that impact decision-making and behavior [61,62]. Additionally, the TDF is underpinned by the COM-B (Capability, Opportunity, Motivation–Behaviour) model, which identifies how these components influence whether a desired behavior will occur [61,63]. The TDF will be used to explore GPs’ capability to engage with the model (eg, knowledge and skills to confidently engage in training or use new models of care), external factors that enable or constrain behavior (eg, opportunities available, resources, time, and environmental context), and the motivation to adopt and sustain these changes (eg, weighing pros and cons, beliefs, and emotional regulation). The TDF and COM-B will ensure that the supports developed as part of the 3 pillars are closely aligned with the needs and challenges of GPs and practice staff, to increase the likelihood of successfully integrating the model into routine practice and improving uptake and sustainability.

This protocol adheres to the Comprehensive Criteria for Reporting Qualitative Research (CCQR) guidelines [64]. The CCQR checklist consists of 14 categories and has been used to ensure comprehensive and transparent reporting of both the qualitative and implementation aspects of our study.

### Setting

EMPHN serves a geographically and demographically diverse region in Victoria, Australia. EMPHN encompasses 12 local government areas (also known as municipalities), including the cities of Boroondara, Whitehorse, Knox, Maroondah, Manningham, Monash, Yarra Ranges, and parts of Banyule and Nillumbik. Approximately 1.62 million people live within the EMPHN catchment, where 441 GP clinics operate [65]. EMPHN plays a pivotal role in coordinating and enhancing primary health care services in this region, with a focus on improving health outcomes, particularly for vulnerable and underserved populations. The network is actively involved in implementing initiatives aimed at reducing health disparities, enhancing chronic disease management, and improving mental health services. PHNs support GPs by offering resources, training, and access to data and tools to help improve patient care. They also facilitate collaboration between GPs, specialists, and allied health providers.

### Part 1: Desktop Audit

A desktop audit will be conducted to determine the current state of GP training and supports for mental health care. The audit will search for available training materials, curricula, guidelines, and resources used in GP training programs. This will include an examination of both formal educational content provided by medical schools and ongoing professional development offerings. The search will be limited to training available to GPs in the EMPHN catchment (including training that is available nationally).

A systematic search of known GP training providers (Royal Australian College of General Practitioners [RACGP], BlackDog, EMHPrac, Australian College of Rural and Remote Medicine [ACRRM], Inside Out, Mind Gardens, the Victorian and Australian government, Mental Health Professional Online Development [MHPOD] Program) and the EMPHN website will be conducted. A comprehensive Google search will also be conducted. The search for each website will use the key concepts of “mental health,” “mental health skills training,” “mental health training” AND “general practitioners,” and “mental health guidelines.” More specific terms related to mental health conditions will also be used (eg, depression, anxiety, domestic/family violence, substance misuse, and eating disorders). Due to the potentially high volume of available training, searches will continue until 3 pages of search results have passed with no relevant results. Identification of publicly available documents related to mental health quality improvement initiatives in general practices within the EMPHN catchment area will also be searched via Google. This search will specifically target mental health standard operating procedures, Plan-Do-Study-Act (PDSA) cycles, quality improvement plans, and mental health audits. These documents represent formal processes and strategies that practices may use to improve their mental health care delivery. The search will use combinations of keywords such as “mental health,” “quality improvement,” “PDSA,” “audit,” and “standard operating procedure.”

The searches will be conducted from inception to 2024. Only training in English language will be included. Training will need to be accessible to GPs working in the EMPHN catchment and will be limited to training offered in Australia. All searches were conducted within a 1-week period in December 2024 to ensure consistency in available online content. The online survey (described in part 2) will also elicit information about GP mental health training.

### Part 2: Online Survey

A 30-minute online survey will be sent to GPs, practice nurses, practice managers, and mental health service managers working in GP clinics within the EMPHN catchment. A target of over 100 responses will be sought via direct email, noticeboard posts, and advertisement on the EMPHN website. Advertisements will include a link to, or an attachment of, the plain language statement and consent information. Surveys will be completed digitally (via REDCap [Research Electronic Data Capture]) [66,67], although, if required, the surveys can be completed via pen and paper and returned via email, fax, or post. The survey will collect demographic information, including information about length of practice, qualifications, and current level of mental health training. Survey questions (see Multimedia Appendix 1) will be quantitative and will ask about current mental health practices and include a set of questions that relate to the definitions of the 14 domains of the TDF as per Michie et al [61,63]. Specifically, using a 7-point Likert scale ranging from “1=strongly disagree” to “7=strongly agree,” participants will be asked to endorse their agreement with statements such as “Addressing mental health issues strengthens the doctor-patient relationship” (Beliefs About Consequences domain) and “I find mental health consultations emotionally draining” (Emotions domain).

The survey was developed through a collaborative process involving our research team’s expertise in mental health care, general practice, and implementation science. Definitions of each of the TDF domains as they relate to mental health care were developed in consultation with the research team (see Multimedia Appendix 2). The questions were then formulated to align with the 14 domains of the TDF definitions, ensuring comprehensive coverage of potential barriers and enablers of implementing mental health care in general practice. To capture demographic information and current mental health practices, we adapted items from a survey used in similar research contexts over the past 15 years. This survey was developed from other validated surveys with permissions by the original developers [68-70]. This approach allowed us to build on established measures while tailoring the content to our specific research questions. Although we did not formally validate the entire survey, we conducted an internal review whereby each of us critically assessed each item for clarity, relevance, and alignment with the TDF domains. This process included multiple iterations of refinement based on team feedback.

### Part 3: Semistructured Interviews

Semistructured interviews will be conducted with up to 20 GPs working in the EMPHN catchment. Practice nurses will also be invited to be interviewed; however, the priority will be gathering the perspectives of GPs. GPs will be recruited through purposive sampling to ensure a diverse representation of practice sizes, locations, genders, and years of clinical practice. Invitations to participate will (1) appear at the end of the online survey (detailed in part 2), (2) be sent via direct email to GPs and practice nurses, and (3) be posted on online noticeboards and the EMPHN website. The direct invitations and notices will include plain language information and a copy of the consent form. Interested participants will be asked to complete an online expression of interest form or to email the research team directly. The research team will then call or email the participant to arrange the interview at a mutually convenient time. Potential participants who have not previously completed the online survey will be asked to complete the demographic portion of the survey only, prior to attending the interview. Verbal consent will be audio-recorded prior to the commencement of the interview.

Interviews will be conducted via telephone, video call (Zoom), or in person. Each interview will last approximately 60 minutes. During the interview, up to 3 vignettes will be presented to the participant to facilitate discussion around how they usually provide mental health care, potential areas for improvement, and how the Link-me+EMPHN Mental Health Model of Care supports might fit in with their practice. Each vignette will focus on 1 of 3 different mental health diagnoses: depression, ADHD, and anxiety (see Multimedia Appendix 3). The interview questions and prompts will be informed by definitions of the 14 domains of the TDF as per Michie et al [61,63].

### Part 4: Co-Designing Link-me+ With Providers and Consumers

To ensure that the implementation and refinement of the Link-me+ service is grounded in the experiences, needs, and preferences of general practice patients with lived experience of mental health issues and mental health care, up to 2 co-design sessions and 4 expert working group workshops will be facilitated.

Each co-design session will involve 5-10 participants. These participants will be recruited from an existing network of people with lived experience of mental health conditions and mental health help-seeking in general practice [71]. Participants will be provided with detailed information about the study and the co-design process prior to their participation. Participants in these co-design sessions have previously provided consent as part of the network members’ participation in co-design activities [71]. Each co-design session will be facilitated by a co-design expert who also has a professional background in developing new and evidence-based models of mental health care for the primary care setting. The sessions will be structured to encourage open dialogue and the sharing of personal experiences. They will begin by introducing the purpose of the Link-me+ model of care and the role of participants in shaping its design. Participants will then be guided through a series of structured activities designed to elicit their priorities for how they would like to use Link-me+ when visiting their GP. These activities may include brainstorming sessions, group discussions, and the creation of visual representations (eg, journey mapping or storyboarding) of their ideal interaction with the Link-me+ model of care (ie, the prognostic tool and tailored treatment options). Discussions during the co-design sessions will be audio-recorded and transcribed verbatim.

Four hybrid (in-person and videoconference via Zoom) expert working group sessions will also be facilitated in the style of co-design. The working group consists of 15 people, including GPs, practice managers, nurses, service managers, researchers, and EMPHN staff. Each session will run for 6 hours and aims to determine how all 3 pillars of the Link-me+EMPHN Mental Health Model of Care will be implemented in practice. The sessions will be structured to (1) provide an overview of each pillar and its operationalization (eg, who is involved and where) and (2) facilitate a discussion to identify key priorities, less critical aspects, and potential additions for consideration. Throughout the sessions, participants will use sticky notes on journey boards to independently write down their perspectives. These will be collected by researchers and the EMPHN team. The sessions will also be audio-recorded and transcribed.

### Part 5: GP Clinic Simulation

Think-aloud simulation sessions with up to 12 GPs, patients, and Care Navigators from the previous Link-me RCT [50] will be conducted using a prototype of the Link-me+ platform in a GP clinic setting. Some simulations may involve a GP-patient participant pair and/or a GP with a patient actor or a patient with a GP actor. Other simulations will be a participant completing the workflow with a member of the research team. The simulations will take place at the Digital Health Validitron SimLab at Melbourne Connect, University of Melbourne, or via videoconference (Zoom) where necessary. The Validitron is run by a multidisciplinary team of clinicians and research experts in digital health implementation, user design and human factors, and evaluation [72].

GPs working in the EMPHN catchment area will be invited to take part in the simulations via email, post, and online noticeboard advertisements asking them to email the research team to express interest. GPs who complete the online survey (detailed in part 2) will also be invited to take part at the completion of the survey. Patient participants will be recruited from the existing pool of people with lived experience of mental health problems used for the co-design sessions (part 4) [71]. Previous Care Navigators will be invited via email or telephone call. The Validitron team has access to a pool of actors who they will recruit as needed.

Different simulated clinic scenarios, based on insights from parts 1 to 4 of this formative research, will be created to mirror situations GPs, patients, and Care Navigators may face in practice when using the Link-me+EMPHN Mental Health Model of Care supports. Scenarios may include tasks such as completing the Link-me+ prognostic tool, diagnosing and assessing a patient’s mental health status, selecting appropriate interventions, or coordinating care with mental health specialists and the Care Navigator. During the simulations, GPs, patients, and Care Navigators will be told to verbalize their thoughts in real time as they navigate a simulated version of the Link-me+ platform. For GPs and Care Navigators, this may include using the Link-me+EMPHN Mental Health Model of Care supports (eg, SupportConnect). All participants will be asked to verbalize their decision-making processes, challenges, and overall experience with the system. Each simulation session will last approximately 60 minutes. For in-person simulation sessions, participants will be observed by the research team through a 2-way mirror at the Validitron, and field notes will be taken. All in-person and videoconference sessions will also be audio-recorded and transcribed.

### Data Analysis

#### Desktop Audit

Relevant data will be extracted from the search results into a purpose-built Microsoft Excel spreadsheet. Qualitative content analysis will be conducted to systematically categorize and interpret the extracted data. This will involve identifying the content of the available GP training and extracting the key elements according to relevance to the 14 TDF domains. For example, training that focuses on increasing understanding of symptom recognition, mental health comorbidities, and available community support services will be mapped to the “Knowledge” domain, while training that reinforces GPs’ identity as holistic care providers will be coded under the “Social/Professional Role and Identity” domain. In addition to categorizing the content, quantitative content analysis will be used to count the frequency of particular elements. For instance, how often key areas such as symptom recognition, mental health comorbidities, or community support services are covered in the training programs. This will provide insight into how well-represented different domains are and highlight any gaps in training coverage.

#### Online Survey

Descriptive statistics for the demographic and TDF items will be calculated using Stata 15. Responses related to the TDF domains will be analyzed by summarizing and categorizing the data under the corresponding domains. This approach will allow for the identification of trends, potential barriers, and gaps within each domain.

#### Semistructured Interviews

The qualitative analysis will employ a dual approach: content analysis using the TDF framework definitions developed by the research team (Multimedia Appendix 2) and thematic analysis as described by Braun and Clarke [73]. The content analysis will be conducted to identify gaps in the current provision of mental health care. Two researchers will independently familiarize themselves with the data through repeated readings of the transcripts. Relevant sections will be coded according to the TDF domains, followed by a frequency count of each reference to a domain. The coding process will be discussed with the research team at regular intervals. Adjustments to definitions and coding will be made as necessary to ensure that the data are appropriately categorized within the framework.

Thematic analysis [73] will also be conducted on the interview transcripts to explore the COM-B elements surrounding the current provision of mental health care and participant suggestions for improvements. Deidentified transcripts will be read and reread by 2 researchers, and any patterns in the data relating to capability, opportunity, motivation, and other influences on GP and practice nurse behaviors will be noted. Identification of what may support future behavior change in mental health care will also be noted. Initial coding for thematic analysis will be generated from the notes. Ongoing coding will be iterative and discussed regularly within the research team until final themes are decided upon.

#### Co-Designing Link-me+ With Providers and Consumers

The data collected as part of the co-design sessions and the expert working groups will be analyzed thematically. In particular, the data will be coded according to challenges, user needs, barriers, and potential solutions to implementation and participants' priorities for the Link-me+ model of care (eg, improving accessibility and user experience with the process of completing the prognostic tool). For both the co-design sessions and expert working group workshops, a summary of the most important findings from each session will be collated and will include key themes, insights, and any actions. The findings will be used to inform the implementation and integration of the Link-me+EMPHN Mental Health Model of Care in practice to ensure it aligns with the needs and expectations of its end users.

#### GP Clinic Simulation

Qualitative data from the simulation sessions will be analyzed using an inductive thematic analysis approach [73]. The coding will focus on identifying common patterns in both GPs’ and patients’ thought processes, challenges encountered, and features of the platform that facilitated or hindered their use of the Link-me+ tool and Link-me+EMPHN Mental Health Model of Care supports. Special attention will be given to identifying usability issues, areas of confusion, and potential improvements. The findings from this analysis will be used to refine the Link-me+ platform, ensuring it aligns with the workflows and needs of GPs and Care Navigators as well as the preferences and experience of patients.

#### Data Triangulation

Data from the desktop audit, semistructured interviews, and online survey will be triangulated to enhance the validity and reliability of the findings. Comparing and contrasting information obtained from these 3 sources will allow for a more comprehensive and nuanced understanding of the factors influencing the use of the Link-me+EMPHN Mental Health Model of Care supports and Link-me+ so that it is optimized for implementation into practice [74]. Triangulation will also enable a systematic assessment of whether current training adequately addresses each behavioral determinant essential for providing mental health care and provide insight into which domains should be focused on to support GPs as part of the Link-me+EMPHN Mental Health Model of Care [74]. Triangulation will involve using the content analysis of each source and looking to identify (1) convergence of findings, where all three sources indicate the same or similar gaps and/or enablers; (2) discrepancies between what is provided in the audit and GPs’ expressed needs in the surveys and interviews; and (3) complementarity findings (eg, the desktop audit may show the types and frequency of mental health training available to GPs, while the survey could quantify GPs’ participation rates in these trainings. The interviews could then provide in-depth insights into GPs’ experiences with the training, including what they found most useful and what challenges they faced in applying the knowledge in practice. Together, these methods provide a more complete picture of the training landscape, from availability to uptake to practical application [74].

#### Rigor

Rigor for the whole of the formative research has been ensured using the gold standard evaluative criteria for trustworthiness as suggested by Lincoln and Guba [75]: credibility, transferability, dependability, and confirmability. Credibility will be established through data source triangulation, involving participants with diverse backgrounds and experiences across multiple general practices. The relevant multidisciplinary expertise of the research team, including backgrounds in neuroscience, psychology, general practice, pharmacy, mental health services research, and lived experience research, will support confirmability. This diverse perspective will be applied during data analysis, which will be conducted primarily by AC and supported by the authors and a research assistant with expertise in interviewing general practice stakeholders. Confirmability will also be enhanced by providing detailed participant quotes to support the interpretation of data. Transferability will be supported by thoroughly describing the sample in reports and research publications, allowing for potential replication. The sample, which includes GPs, nurses, and practice staff from various general practices, will provide an in-depth analysis of mental health care provision in the Australian general practice context. Dependability will be addressed through documentation of the data gathering and analysis processes, including a detailed protocol, digital notes, meeting minutes, and the publication of study outcomes. The methodology for this study builds upon well-established qualitative research practices and aligns with the theoretical frameworks of the TDF and COM-B model.

### Ethical Considerations

Ethical approval has been obtained from the University of Melbourne Human Research Ethics Committee (HREC ID 29143) for all parts of this research. Informed consent will be obtained from all participants before the commencement of all parts of this formative research with the exception of the desktop audit search results. A well-established distress protocol, in use by the research team for over a decade, will be in place to manage any potential participant distress during the study. This protocol outlines clear procedures for researchers to follow if a participant experiences emotional distress, ensuring prompt and appropriate support is provided. All interview, co-design, and clinic simulation data will be deidentified during the transcription process. Participants will be assigned unique identifiers, and any potentially identifying information will be removed from the transcripts. The online survey is anonymous and does not ask for identifying information. All data will be stored securely on password-protected servers accessible only to the research team. Audio recordings will be deleted after transcription and verification. Interview, co-design, and simulation participants will be compensated for their time and contribution to the study with a Aus $100 (approximately US $66.96) gift card. Respondents to the online survey will go into a randomized draw to win 1 of 10 Aus $100 (approximately US $66.96) gift cards.

## Results

A systematic search of the RACGP CPD learning, MHPOD, BlackDog, EMHPrac, ACRRM, Inside Out, EMPHN, Mind Gardens, and the Victorian and Australian government websites was conducted using relevant keywords (eg, mental health, mental health guidelines, domestic/family violence, substance misuse, and eating disorders) in November 2024. A Google search was also performed in November 2024. A total of 270 GP training opportunities were found. As only the learning objectives for each training course were readily available, these have been extracted for coding to the 14 domains of the TDF. As of June 24, 2025, a total of 30 people have completed the online survey. The survey remained open until the end of June 2025. The majority of participants were female (n=25, 84%), aged 51-60 years (n=12, 40%), and GPs (n=15, 50%) or practice nurses (n=10, 34%). The average time spent working in general practice was 13.65 years. Fourteen interviews with GPs have been completed with recruitment continuing until June 2025. Thematic analysis of the interview transcripts has commenced. Co-design and GP simulations sessions were completed by July 2025.

## Discussion

### Anticipated Findings

The Link-me+EMPHN Mental Health Model of Care is an innovative model that aims to build GP capacity and support them to provide better mental health care for their patients, while fostering patient self-awareness and ownership of care. This formative research aims to identify knowledge gaps in current GP mental health training as well as barriers to and enablers of the implementation of the Link-me+EMPHN Mental Health Model of Care in routine practice by GPs and other practice staff. Potential barriers are likely to include systemic and practice-level challenges, and enablers will include potential catalysts for behavior change among GPs and clinic staff.

Implementing models of care in general practice can be challenging due to a range of systemic and practice barriers including time constraints, high patient demand, and the complexity of addressing diverse health needs [76]. Integration of new models of care also requires additional coordination, training, documentation, collaboration with other services, and the need for behavioral shifts in practice which can impact GP and clinic staff buy-in [77]. To ensure the successful implementation and integration of the Link-me+EMPHN Mental Health Model of Care supports, this formative research will investigate catalysts for GP and clinic staff behavior change by collecting and analyzing data from multiple sources and a variety of stakeholders.

The findings from this formative research will inform potential optimizations of the implementation strategy. These could include tailoring the strategy to address specific barriers identified in different practice settings, developing targeted training programs to address skill gaps, adjusting the model to better align with existing practice workflows, and enhancing support systems for practices during the implementation process. While the core evidence-based components of Link-me+ will remain intact, the formative review may lead to adaptations in how the model is presented, integrated, or supplemented within the broader Link-me+EMPHN Mental Health Model of Care. Such adaptations could include expanding resources for managing comorbidities, incorporating additional assessment modules, refining the care navigation process, or developing supplementary materials to address frequently encountered clinical scenarios.

### Comparison to Prior Work

While previous projects have often focused on single aspects of implementation, such as GP perspectives or organizational barriers, our formative research integrates views from GPs, nurses, practice staff, and mental health consumers, providing a more holistic understanding of the practice environment. For instance, Whitebird et al [78] conducted mixed methods research to identify successful elements for implementing collaborative care for depression in primary care with a focus primarily on organizational readiness and leadership engagement. Our study expands on this by not only considering organizational factors but also individual practitioner skills, attitudes, and behaviors, as well as patient-level considerations.

The Link-me+EMPHN formative research also shares similarities with Snyder et al [79] as both studies employ mixed methods designs and formative evaluation techniques and recognize the importance of tailoring implementation strategies to local contexts. Both studies also contribute to the evolving field of implementation science in mental health care, demonstrating how formative evaluation methods can be adapted to different health care contexts. However, Snyder et al [79] focused on improving suicide prevention practices within a single community mental health clinic, while Link-me+EMPHN addresses a broader spectrum of mental health issues across a network of primary care practices. Additionally, Snyder et al [79] developed a supervisory toolkit as their primary implementation strategy, but our research will offer a more diverse range of strategies due to the complexity of implementing a digital intervention across multiple primary care sites.

The SMART (Systematic Medical Appraisal, Referral, and Treatment) Mental Health programme provides another relevant comparison. SMART Mental Health heavily emphasized the use of mobile-based applications and an electronic decision support system (EDSS) for community health workers and primary care doctors [80]. Our study, while incorporating digital elements, has a broader focus on GP engagement and capacity building. The formative phase for SMART Mental Health included preliminary feasibility testing of their EDSS, providing quantitative data on screening and diagnosis rates [80]. The Link-me component (pillar 3) of the Link-me+EMPHN Model of Care has already undergone testing in 2 RCTs and additional user-testing. Accordingly, our formative phase is more focused on qualitative insights to inform the subsequent implementation strategies. SMART Mental Health was also implemented in rural India, focusing on low-resource settings, while our study is set in an Australian context with a different health care infrastructure.

The findings of a Danish implementation study highlight the extensive implementation work required by care managers, including solving practical problems of location and logistics, engaging GPs, and tailoring collaboration to meet GP preferences [81]. Although our study focuses on the broader implementation of a mental health model of care, we anticipate similar challenges in integrating Care Navigation and other new processes into existing primary care settings.

Collectively, these studies highlight the importance of context-specific approaches in implementation research, showcasing how similar methodological foundations can be applied to address distinct challenges in different mental health care settings. Additionally, research studies specifically addressing the implementation of comprehensive mental health models of care in primary settings are relatively scarce. Although implementation science in health care has advanced [82], there appears to be a need for more focused formative research on integrating complex mental health interventions in primary care. The current project aims to address this gap, potentially providing a template for future formative research in this area. Specifically, Link-me+EMPHN Mental Health Model of Care is an innovative approach to addressing this gap, strengthened by our comprehensive, theoretically grounded approach to its implementation.

### Strengths and Limitations

A key strength of this protocol is its mixed methods approach, combining quantitative surveys with qualitative interviews and desktop audits. This triangulation of data sources will provide rich, contextual insights into the complexities of implementing new mental health care models in general practice. The inclusion of a range of stakeholder perspectives (GPs, practice staff, and mental health consumers) further enhances the comprehensiveness of the study, offering diverse viewpoints on implementation challenges and opportunities. Additionally, the focus on extending and tailoring evidence-based research to optimize adoption, implementation, and successful outcomes in practice addresses a critical gap between research and real-world application. However, potential limitations include the geographical constraint to the EMPHN catchment area, which may limit generalizability to other regions. Additionally, self-selection bias may occur if only practices highly motivated to improve mental health care participate in the study.

### Future Directions

Following the formative research and the development of the GP supports, the Link-me+EMPHN Mental Health Model of Care will be implemented in up to 60 GP clinics in the EMPHN catchment area throughout 2025 to 2027. An evaluation (protocol in development) of the implementation will be conducted guided by the RE-AIM (Reach, Effectiveness, Adoption, Implementation, and Maintenance) [83] and the PRISM (Practical, Robust Implementation, and Sustainability Model) [84] frameworks. The evaluation will systematically assess the effectiveness and sustainability of the mental health model of care and ensure that the model is comprehensive and scalable in real-world settings [83,84].

### Conclusions

This formative research protocol outlines a comprehensive, mixed methods approach to inform the implementation of the Link-me+EMPHN Mental Health Model of Care in general practice settings. Through desktop audits, interviews, surveys, co-design workshops, and simulated practice testing, we aim to identify key gaps in GP mental health training, barriers to and enablers of implementation, and stakeholder perspectives. These methods are designed to yield practical insights that will directly inform the refinement of the model and its implementation strategy. Although the specific findings are yet to emerge from the data, we anticipate that this research will provide a nuanced understanding of the challenges and opportunities in integrating comprehensive mental health interventions in primary care. Therefore, this study contributes to the broader field of implementation science in mental health.

## Appendix

Multimedia Appendix 1 Online survey.

Multimedia Appendix 2 Theoretical domains framework.

Multimedia Appendix 3 Interview schedule and vignettes.

Multimedia Appendix 4 CCQR checklist.

## Abbreviations

ACRRM: Australian College of Rural and Remote Medicine
ADHD: attention-deficit/hyperactivity disorder
CCQR: Comprehensive Criteria for Reporting Qualitative Research
COM-B: Capability, Opportunity, Motivation–Behaviour
EDSS: electronic decision support system
EMPHN: Eastern Melbourne Primary Health Network
GP: general practitioner
IAPT: Improving Access to Psychological Therapies
MHPOD: Mental Health Professional Online Development
PDSA: Plan-Do-Study-Act
PHN: Primary Health Network
PRISM: Practical, Robust Implementation, and Sustainability Model
RACGP: Royal Australian College of General Practitioners
RCT: randomized controlled trial
RE-AIM: Reach, Effectiveness, Adoption, Implementation, and Maintenance
REDCap: Research Electronic Data Capture
SMART: Systematic Medical Appraisal, Referral, and Treatment
TDF: Theoretical Domains Framework

## Data Availability

The data that will be generated as part of this research will not be publicly available to protect the privacy of research participants who will not be asked to provide consent for the public sharing of their data.
