# Supplementary material for: Developing the Link-me+EMPHN Mental Health Model of Care to Improve General Practitioner Capacity for Mental Health Care in Australian Primary Care: Protocol for a Mixed Methods Formative Study
Source: JMIR Res Protoc. 2026 Jan 23;15:e79560. doi: 10.2196/79560 (PMC12881906; doi:10.2196/79560)
Supplement: Multimedia Appendix 1 [file resprot_v15i1e79560_app1.docx]

# Online survey

| Where is the general practice that you currently work  in, located?    See map here:  https://emphn.org.au/about-us/community-snapshot/    or here: EMPHN suburbs | | | | | | | - City of Banyule - City of Knox - City of Monash - Shire of Nillumbik - City of Whittlesea - City of Booroondara - City of Manningham - City of Maroondah - City of Whitehorse - Shire of Mitchell - Shire of Murrindindi - Shire of Yarra Ranges - Other | | | | | |  |  |  |  |
| --- | --- | --- | --- | --- | --- | --- | --- | --- | --- | --- | --- | --- | --- | --- | --- | --- |
| What gender do you identify with? | | | | | | | - Male - Female - Other | | | | | |  |  |  |  |
| What is your age? | | | | | | | ___________ | | | | | |  |  |  |  |
| Are you a | | | | | | | - GP - Practice nurse - Practice manager - Business manager - Service manager, please specify which service   ___________________   - Other, please specify   ________________ | | | | | |  |  |  |  |
| What year did you complete your medical or nursing degree? | | | | | | | __ __ __ __ | | | | | |  |  |  |  |
| What are your qualifications? (Please tick all that apply) | | | | | | | - FRACGP / FACRRM or equivalent - PhD, MD - Bachelor of Nursing - Diploma of Nursing - Masters of Mental Health Nursing - Masters in Mental Health/Psychiatry - Diploma in Mental Health/Psychiatry - Other Masters, Diploma or Certificate, please specify ______________________ - Other, please specify __________________ | | | | | |  |  |  |  |
| Have you done any additional mental health training? | | | | | | | - Level 1 Mental Health Skills Training - Level 2 Focussed Psychological Strategies Skills Training - Mental Health First Aid - Other, please specify __________________ | | | | | |  |  |  |  |
| What other mental health training have you completed? | | | | | | | __ __ __ __ | | | | | |  |  |  |  |
| What country/region did you graduate in?  (Please tick all relevant responses) | | | | | | | - Australia - New Zealand - Asia - United Kingdom - Other, please specify __________________ | | | | | |  |  |  |  |
| How many years have you spent working in general practice? | | | | | | | In Australia: _______ years  Overseas: _______ years | | | | | |  |  |  |  |
| In your current practice, are you: | | | | | | | □ Partner / Principal  □ Associate  □ Employee  □ Other, please specify: __________________________ | | | | | |  |  |  |  |
| What type of billing does your practice use? | | | | | | | - Bulk billing - Mixed billing - Private billing | | | | | |  |  |  |  |
| Which of the following patients do you/does your practice routinely bulk bill?  (Please tick all relevant responses) | | | | | | | - All patients - Pensioners - Non-pension health care card holders - Children - None | | | | | |  |  |  |  |
| In a usual week, what proportion of your patient consultations are: | | | | | | | - <6 mins (Item A) - 6-20 mins (Item B) - 21-40 mins (Item C) - 41+ mins (Item D) | | | | | |  |  |  |  |
| In a usual week, how many hours do you work in general practice (or your general practice workplace)? | | | | | | | - Less than 15 - 16-30 - 31-45 - 46 or more | | | | | |  |  |  |  |
| In what proportion of your adult patients in the past 12 months would you estimate depression and/or anxiety to be a significant part of the clinical picture? | | | | | | | _________ % | | | | | |  |  |  |  |
| In what proportion of your adult patients in the past 12 months would you estimate mental health conditions other than depression and/or anxiety to be a significant part of the clinical picture? | | | | | | | _________ % | | | | | |  |  |  |  |
| How would you describe yourself as a provider of mental health care? | | | | | | | - Novice - I have minimal experience and knowledge in providing mental health care - Beginner - I have some experience but still require significant guidance and support - Intermediate - I am comfortable providing mental health care but occasionally seek assistance - Proficient - I am skilled and confident in providing mental health care, needing little assistance - Expert - I have extensive experience and expertise in providing mental health care and can offer guidance to others | | | | | |  |  |  |  |
| How much of a priority is regularly updating your skills and knowledge in mental health care for you? | | | | | | | - Not a priority at all - Low priority - Moderate priority - High priority - Very high priority | | | | | |  |  |  |  |
| Please indicate what level of training you have undertaken in the following areas:  (Please tick as many responses as apply for each question) | | | | | | | | | | | | |  |  |  |  |
|  | | None | | Self taught | | Lecture / seminar / workshop | Formal qualification | | | | | |  |  |  |  |
| Cognitive behavioural therapy | | □ | | □ | | □ | □ | | | | | |  |  |  |  |
| Interpersonal therapy | | □ | | □ | | □ | □ | | | | | |  |  |  |  |
| Family therapy | | □ | | □ | | □ | □ | | | | | |  |  |  |  |
| Motivational interviewing | | □ | | □ | | □ | □ | | | | | |  |  |  |  |
| Problem solving therapy | | □ | | □ | | □ | □ | | | | | |  |  |  |  |
| Managing patients with mental health symptoms can sometimes be challenging. Please indicate the amount of support you receive from the following sources when managing adult patient mental health. | | | | | | | | | | | | |  |  |  |  |
|  | | | | Never/  Rarely | | Sometimes | Often | | | | | |  |  |  |  |
| Discussing cases with psychiatrist | | | | □ | | □ | □ | | | | | |  |  |  |  |
| Discussing cases with GP colleagues | | | | □ | | □ | □ | | | | | |  |  |  |  |
| Discussing cases with other health professionals (e.g., clinical psychologist) | | | | □ | | □ | □ | | | | | |  |  |  |  |
| Attendance at Balint group | | | | □ | | □ | □ | | | | | |  |  |  |  |
| Professional supervision | | | | □ | | □ | □ | | | | | |  |  |  |  |
| Support from partner, family or friends | | | | □ | | □ | □ | | | | | |  |  |  |  |
| Resources from organisations | | | | □ | | □ | □ | | | | | |  |  |  |  |
| Could you tell us what resources you have used and which organisations they came from: | | | |  | |  | ________________________________________________ | | | | | |  |  |  |  |
| Do you receive/use any other support/s?  Please specify: | | | |  | |  | ________________________________________________ | | | | | |  |  |  |  |
|  | | | |  | |  |  | | | | | |  |  |  |  |
|  | | | |  | |  |  | | | | | |  |  |  |  |
|  | | | |  | |  |  | | | | | |  |  |  |  |
|  | | | |  | |  |  | | | | | |  |  |  |  |
|  | | | |  | |  |  | | | | | |  |  |  |  |
| When seeing adult patients with mental health symptoms in the past 12 months, for what proportion of these patients did you use each of the following strategies? | | | | | | | | | | | | | | | |  |
| I provided psychoeducation | | | | | - None - Very few - About a quarter - About half - About three-quarters - Almost all/all | | | | | | | | | | |  |
| I provided the patient with educational resources | | | | | As above | | | | | | | | | | |  |
| I taught the patient meditation and/or relaxation techniques | | | | | As above | | | | | | | | | | |  |
| I gave advice on getting a good night's sleep | | | | | As above | | | | | | |  | | | |  |
| I provided supportive counselling | | | | | As above | | | | | | | | | | |  |
| I taught the patient structured problem solving | | | | | As above | | | | | | | | | | |  |
| I used hypnosis with the patient | | | | | As above | | | | | | | | | | |  |
| I prescribed antidepressants | | | | | As above | | | | | | | | | | |  |
| I prescribed benzodiazepines | | | | | As above | | | | | | | | | | |  |
| I prescribed other medications (for example, antipsychotics, stimulants) | | | | | As above | | | | | | | | | | |  |
| I spoke to patient/s about stopping their mental health medication/s | | | | | As above | | | | | | | | | | |  |
| I used the Initial Assessment and Referral Decision Support Tool (IAR-DST) | | | | | As above | | | | | | | | | | |  |
| I encouraged the patient to exercise | | | | | As above | | | | | | | | | | |  |
| I provided family or marital counselling | | | | | As above | | | | | | | | | | |  |
| I used cognitive behavioural therapy | | | | | As above | | | | | | | | | | |  |
| I encouraged patient/s to change lifestyle factors (for example, exercise, sleep hygiene, diet) | | | | | As above | | | | | | | | | | |  |
| I asked about drug and alcohol intake | | | | | As above | | | | | | | | | | |  |
| I provided the patient with drug and alcohol counselling | | | | | As above | | | | | | | | | | |  |
| I gave advice on getting a good night’s sleep | | | | | As above | | | | | | | | | | |  |
| I told patient/s about mental health services they might use | | | | | As above | | | | | | | | | | |  |
| Other, please specify  _________________________ | | | | | As above | | | | | | | | | | |  |
| Other, please specify  _________________________ | | | | | As above | | | | | | | | | | |  |
| Other, please specify  _________________________ | | | | | As above | | | | | | | | | | |  |
| Are there any other strategies you used with patient/s for their mental health in the past 12 months? Please specify | | | | | _________________________________________ | | | | | | | | | | |  |
| The following questions are about how you feel about your current practices when providing mental health care. | | | | | | | | | | | | | | |  |  |
|  | | Strongly disagree | | Disagree | | Somewhat disagree | | | Neither disagree or agree | Somewhat agree | Agree | | Strongly agree | | | |
| It is part of my role to identify mental ill-health | | □ | | □ | | □ | | | □ | □ | □ | | □ | | | |
| I am familiar with how to speak with patients about their mental health | | □ | | □ | | □ | | | □ | □ | □ | | □ | | | |
| I have been trained in discussing patients’ mental health care goals with them to enable me to plan their treatment | | □ | | □ | | □ | | | □ | □ | □ | | □ | | | |
| I am prepared for my discussions with patients and their families/carers about their mental health by reviewing their medical information beforehand | | □ | | □ | | □ | | | □ | □ | □ | | □ | | | |
| I check in regularly with patients whether I have understood their perspectives on their mental health symptoms | | □ | | □ | | □ | | | □ | □ | □ | | □ | | | |
| I am confident in identifying when mental ill-health symptoms are mild, moderate or severe | | □ | | □ | | □ | | | □ | □ | □ | | □ | | | |
| I feel confident about speaking to my patients about their mental health | | □ | | □ | | □ | | | □ | □ | □ | | □ | | | |
| I have the skills and resources necessary to manage patients with severe mental health symptoms | |  | |  | |  | | |  |  |  | |  | | | |
| I intend to prioritise mental health care in my practice more | |  | |  | |  | | |  |  |  | |  | | | |
| I believe that my efforts in mental health care will lead to positive changes for patients | |  | |  | |  | | |  |  |  | |  | | | |
| I plan to adopt new mental health care practices if evidence supports their effectiveness | |  | |  | |  | | |  |  |  | |  | | | |
| Our practice keeps patient medical records up-to-date regarding their mental health | |  | |  | |  | | |  |  |  | |  | | | |
| My clinic environment supports the use of new tools for mental health care | |  | |  | |  | | |  |  |  | |  | | | |
| It is my responsibility to connect patients with mental health resources | |  | |  | |  | | |  |  |  | |  | | | |
| I am effective at prioritising patient concerns when addressing mental health symptoms without assistance | |  | |  | |  | | |  |  |  | |  | | | |
| I am familiar with the local mental health care pathways and referral processes in my area | |  | |  | |  | | |  |  |  | |  | | | |
| I am familiar with the local mental health care pathways and referral processes in my area | |  | |  | |  | | |  |  |  | |  | | | |
| Collaborating with my peers or other mental health professionals enhances my ability to provide effective patient care | |  | |  | |  | | |  |  |  | |  | | | |
| Acting on the advice of my peers or other mental health professionals can enhance the quality of care I provide | |  | |  | |  | | |  |  |  | |  | | | |
| I am confident in my ability to manage mental health cases without additional support | |  | |  | |  | | |  |  |  | |  | | | |
| I seek support from local GPs or other mental health professionals when faced with complex clinical situations | |  | |  | |  | | |  |  |  | |  | | | |
| I prioritise updating my skills and knowledge on mental health care management | |  | |  | |  | | |  |  |  | |  | | | |
| I have easy access to resources and networks that support decision-making in patient care | |  | |  | |  | | |  |  |  | |  | | | |
| Offering mental health care in my practice requires more resources than my practice currently has available | |  | |  | |  | | |  |  |  | |  | | | |
| I feel reassured when I can consult with peers or other mental health professionals regarding complex cases | |  | |  | |  | | |  |  |  | |  | | | |
| I am optimistic that my practice can improve its mental health care delivery | |  | |  | |  | | |  |  |  | |  | | | |
| I regularly review and adapt my (or my practices)approach to mental health care based on patient feedback and outcomes | |  | |  | |  | | |  |  |  | |  | | | |
| Decision-making in mental health care is straightforward and well-supported by our clinic | |  | |  | |  | | |  |  |  | |  | | | |
| I easily recall the steps needed to manage a mental health condition during patient consultations | |  | |  | |  | | |  |  |  | |  | | | |
| I find mental health consultations emotionally draining | |  | |  | |  | | |  |  |  | |  | | | |
| I feel a sense of fulfillment when I successfully help patients with mental health issues | |  | |  | |  | | |  |  |  | |  | | | |
| My practice's capacity to deliver mental health care is limited by time constraints | |  | |  | |  | | |  |  |  | |  | | | |
| Addressing mental health issues strengthens the doctor-patient relationship | |  | |  | |  | | |  |  |  | |  | | | |
| I worry that if mental health care is not effectively managed, it could lead to negative outcomes for the patient | |  | |  | |  | | |  |  |  | |  | | | |
| I am motivated to improve my mental health care skills and regularly set goals to enhance patient outcomes in this area | |  | |  | |  | | |  |  |  | |  | | | |
| My clinic prioritises mental health care as part of our commitment to patient-centred care | |  | |  | |  | | |  |  |  | |  | | | |
| My clinic and I face challenges in achieving my mental health care goals due to time constraints, competing responsibilities, or lack of resources | |  | |  | |  | | |  |  |  | |  | | | |
| There are mechanisms in place within my mental health practices (e.g., supervision, peer support) that reinforce the importance of providing mental health care | |  | |  | |  | | |  |  |  | |  | | | |
| Which statement is more true for you: | |  | | I tailor my management to needs and wishes of the patient  A mix of both  I tailor my management to the patient’s diagnosis | | | | | | | | |  | | | |
| The next few questions ask about your thoughts on the mental health care that currently happens in your clinic.  You may provide as much information as you would like and you are welcome to use dotpoints. | | | | | | | | | | | | |  | | | |
| Eastern Melbourne Primary Health Network (EMPHN) is one of the major commissioners of Commonwealth-funded health care services in Melbourne, with responsibility for improving the primary healthcare system. They  facilitate health system improvements for people in eastern and north-eastern Melbourne. | |  | | ______________________________________________________________________________________________________ | | | | | | | | |  | | | |
| Regarding patient visits, can you briefly describe what happens in a usual appointment where mental health may be discussed? Is it usually the focus of the appointment, or not? | |  | | ______________________________________________________________________________________________________ | | | | | | | | |  | | | |
| Regarding patient visits can you briefly describe what happens during a health assessment (or other interaction) where mental health may have been discussed? How do you approach these situations and how is this information communicated with GPs and other practice staff? | |  | | ______________________________________________________________________________________________________ | | | | | | | | |  | | | |
| Regarding patient visits, what interactions do you have with patients that help you pick up on signs of anxiety or other concerns? How do you typically communicate this information to the GP or other healthcare team members? | |  | | ______________________________________________________________________________________________________ | | | | | | | | |  | | | |
| Regarding patient visits, what interactions do you have with patients that help you pick up on signs of anxiety or other concerns? How do you typically communicate this information to the GP or other healthcare team members? | | \|  \| ______________________________________________________________________________________________________ \| \| --- \| --- \| | | | | | | | | | | |  | | | |
| What areas are challenging for you (or your practice) when diagnosing, treating and managing mental health? | | \|  \| ______________________________________________________________________________________________________ \| \| --- \| --- \| | | | | | | | | | | |  | | | |
| What do you and/or your practice need to enhance mental health training and/or increase confidence in providing mental health care? | | \|  \| ______________________________________________________________________________________________________ \| \| --- \| --- \| | | | | | | | | | | |  | | | |
| What patient outcomes have you seen from the mental health care practices in your clinic?  In what ways do these outcomes impact your or your practices future provision of care? | | \|  \| ______________________________________________________________________________________________________ \| \| --- \| --- \| | | | | | | | | | | |  | | | |
| Have you and/or your clinic used a "Plan Do Study Act (PDSA)" system, or taken part in clinic audits/quality improvement to implement or strengthen mental health care?  If so, what were they and in what ways did you find them helpful or unhelpful? | | \|  \| ______________________________________________________________________________________________________ \| \| --- \| --- \| | | | | | | | | | | | | | | |
| Can you describe how you (and your clinic) currently stay informed about mental health clinical guidelines or local mental health care pathways when managing patient care? | | \|  \| ______________________________________________________________________________________________________ \| \| --- \| --- \| | | | | | | | | | | |  | | | |
| Finally, is there anything that you would like to add about mental health care in your practice?  For example, what works well, what doesn't work well, etc. | | \|  \| ______________________________________________________________________________________________________ \| \| --- \| --- \| | | | | | | | | | | |  | | | |
|  | |  | |  | |  | | |  |  |  | |  | | | |
