# Supplementary material for: Developing the Link-me+EMPHN Mental Health Model of Care to Improve General Practitioner Capacity for Mental Health Care in Australian Primary Care: Protocol for a Mixed Methods Formative Study
Source: JMIR Res Protoc. 2026 Jan 23;15:e79560. doi: 10.2196/79560 (PMC12881906; doi:10.2196/79560)
Supplement: Multimedia Appendix 2 [file resprot_v15i1e79560_app2.docx]

# TDF definitions

The definitions of the 14 domains as they pertain to the Link-me+EMPHN Model are as follows:

1. Knowledge: What gaps exist in understanding common mental health disorders or therapeutic approaches?
2. Skills: Are GPs given adequate skills to assess, diagnose, and manage mental health conditions in primary care settings? Are there gaps in crisis management, risk assessment, or communication?
3. Social/professional role and identity: How do GPs perceive their role in delivering mental health care?
4. Beliefs about capabilities: Does training help GPs to feel confident in their ability to manage complex mental health cases?
5. Environmental context and resources: Does the current training reflect the real-world environment of primary care?
6. Memory, attention, and decision processes: Are GPs trained to make quick, informed decisions about mental health care under pressure?
7. Behavioural regulation: Are there mechanisms in place to ensure that GPs continuously apply what they’ve learned in their daily practice? What monitoring or feedback loops are missing?
8. Beliefs about consequences: What increases GP motivation to engage in good mental health care?
9. Optimism: Does training instil a sense of hope or efficacy in GPs? Is there an emphasis on patient recovery?
10. Intentions: Are GPs encouraged to set goals, intentions or action plans?
11. Goals: Are GPs given benchmarks or other measurable goals?
12. Reinforcement: Are there systems for providing feedback, recognition, or rewards for GPs who successfully implement mental health strategies?
13. Emotion: Do the materials consider the potential for GPs to feel overwhelmed, anxious, or empathetically exhausted when managing mental health cases? Are coping strategies included?
14. Social influences: Does the training encourage GPs to consult with mental health professionals or rely on a support network?
