# Supplementary material for: Developing the Link-me+EMPHN Mental Health Model of Care to Improve General Practitioner Capacity for Mental Health Care in Australian Primary Care: Protocol for a Mixed Methods Formative Study
Source: JMIR Res Protoc. 2026 Jan 23;15:e79560. doi: 10.2196/79560 (PMC12881906; doi:10.2196/79560)
Supplement: Multimedia Appendix 3 [file resprot_v15i1e79560_app3.docx]

# Interview schedule and vignettes

| Firstly, can you tell me about your role in mental health care?  ***Prompts and/or follow-ups:***   - *Do you think it should be part of your role?* - *What other role/s should be available to assist with mental health care in general practice?* - *What motivates you to seek advice or support from your peers or other mental health professionals when faced with mental health care decisions?* - *What challenges do you face when seeing patients with MH issues? How do you work around these challenges?* - *What training are you aware of but haven’t accessed and why not?* - *What would increase your confidence in providing mental health care?* - *What are the difficulties you face in providing* ***tailored*** *mental health care?*    - *What would help/support you to do this better?* - *How confident do you feel in recognizing mental ill-health?*   - *What would help you feel more confident?* - *What patient outcomes have you seen from your mental health care practices?* - *What do you think is the most/least helpful in the care that you can provide? Why? In what ways do these outcomes impact your future provision of care?* - *What factors or experiences have shaped this view?* |
| --- |
| Thank you. Moving on now, I’d like to read a vignette to you and I’ll pop it in the chat for you as well.  John Smith is a 30 year old married male who feels down most of the time. He experiences frequent, intrusive thoughts that he is not good enough, despite personal and professional successes. He tries to overcompensate for his thoughts by taking on more than he can handle, which leads to failure and furthers his feelings of inadequacy. His wife suggests that he seek help after finding him crying. John tells you:  “I feel pretty worthless and have felt that way for a long time - for over a couple of years. It feels normal to be down. Sometimes I start to feel better, but it never lasts - I feel bad about my life most of the time. I don’t think that I’ll amount to anything and l might never be successful at work. I feel exhausted all of the time. I have trouble sleeping. I’ve been waking up in the middle of the night and can’t stop thinking about all of my failures. I’ve lost weight in the past few months. Food just doesn’t taste as good as it used to. I feel hopeless and often have trouble concentrating when I’m with my family and friends. Maybe I’m just pessimistic.”  What are your initials thoughts? How would you explore John’s symptoms with him?  *(Would this differ if he was a new patient? If you had seen him in the past?)*  ***Follow up questions and/or prompts:***   - *How does mental health usually come up in an appointment?* - *Can you tell me what happens if/when it does?* - *When you are with a patient and talking about their mental health, what feelings arise for you?* - *What resources have you used or do you know of to support your with mental health care?* - *What resources from your PHN or other organisations are you aware of and/or do you use for your own knowledge or your patients’?*   Great, thank you. Is there anything else you would like to add before we move on? |
| Thank you.  Thinking about a different patient now:  Susan is a 36 year old female, currently living at home with her husband of 11 years and their 2 year old daughter. She is experiencing increased levels of stress as she tries to balance the challenges of parenting with the demands of her career as a dietician. Susan reports a longstanding history with poor attention. She was identified in grade school as requiring special accommodations and support from her teachers, but was never diagnosed with a confirmed learning disability. Her difficulties with focusing and time management have continued into adulthood, affecting her ability to effectively manage her finances and to balance her work/family time relationship. She has been able to cope by creating “pressure” to engage her focus, but this has resulted in symptoms of anxiety, depression, feelings of guilt, and loss of self-esteem.  Susan is concerned with perceived underachievement and inefficiency at her place of employment. She struggles with procrastination, and has difficulty with organization. Due to the increased demands and responsibilities she now faces in the home, Susan finds that she is not able to focus on work-related duties the way she used to. As a result, she has been disappointed by her recent performance at her place of work.  Susan is an intelligent and engaging communicator, but she feels overwhelmed by her current responsibilities and worries that she is not performing to her real potential. She sometimes feel paralyzed by stress, and reacts by retreating into herself rather than by asking for help.  She has been on an antidepressant for 8 years from her GP. She demonstrates no hyperactivity, but is challenged to stay focused to even read a book.  What are your initials thoughts? How would you explore Susan’s concerns with her?  ***Prompts and follow-up questions as above.*** |
| IF TIME AVAILABLE  Mike is a 20 year-old who reports to you that he feels depressed and is experiencing a significant amount of stress about school, noting that he’ll “probably flunk out.” He spends much of his day in his dorm room playing video games and has a hard time identifying what, if anything, is enjoyable in a typical day. He rarely attends class and has avoided reaching out to his professors to try to salvage his grades this semester. Mike has always been a self-described shy person and has had a very small and cohesive group of friends from primary through high school. Notably, his level of stress significantly amplified when he began university. You learn that when meeting new people, he has a hard time concentrating on the interaction because he is busy worrying about what they will think of him – he assumes they will find him “dumb,” “boring,” or a “loser.” When he loses his concentration, he stutters, is at a loss for words, and starts to sweat, which only serves to make him feel more uneasy. After the interaction, he replays the conversation over and over again, focusing on the “stupid” things he said. Similarly, he has a long-standing history of being uncomfortable with authority figures and has had a hard time raising his hand in class and approaching teachers. Since starting university, he has been isolating more, turning down invitations from his roommate to go eat or hang out, ignoring his phone when it rings, and habitually skipping class. His concerns about how others view him are what drive him to engage in these avoidance behaviors.  How would you explore Mike’s concerns with him?  ***Prompts and follow-up questions as above.*** |
