# Supplementary material for: Developing the Link-me+EMPHN Mental Health Model of Care to Improve General Practitioner Capacity for Mental Health Care in Australian Primary Care: Protocol for a Mixed Methods Formative Study
Source: JMIR Res Protoc. 2026 Jan 23;15:e79560. doi: 10.2196/79560 (PMC12881906; doi:10.2196/79560)
Supplement: Multimedia Appendix 4 [file resprot_v15i1e79560_app4.docx]

Comprehensive Criteria for Reporting Qualitative Research (CCQR)

| Topic | Description | Page # |
| --- | --- | --- |
| Title of the paper | Draws and attracts the reader and entails precisely what the paper covers; must be of relevance to intext content and precise | 1 |
|  | Include types of study | 1 |
| Abstract | Key elements of research including background, introduction, purpose, methodology, results, and conclusion | 1-2 |
|  | Under the abstract, include keywords | 2 |
| Introduction | Aim/objectives and purpose of the study | 6 |
|  | Relevance/justification of research question/connection to existing knowledge | 3-6 |
|  | Problem statement/rationale | 6-8 |
|  | Describing existing knowledge | 4-5 |
| Methodology | Research design/theoretical framework/research paradigm | 9-10 |
|  | Rationale for chosen methodology | 9-10 |
|  | Study population (characteristics, sample size, inclusion, and exclusion criteria) | 11-15 |
|  | Study site | 11 |
|  | Sampling technique | 11-15 |
|  | Researchers and participants relationship | 18-19 |
|  | Data collection technique/methods (e.g., in-depth interviews, focus group discussions (FGDs), observation, memos, field notes, audio recording, and video recording) | 11-15 |
|  | Data collection process (period of data collection and timing of interview) | 15 |
|  | Data collection tools (interview guide) | Appendix |
|  | Data analysis and data management (transcription, software, coding, theme generation, coding tree, number of data coders, data security, and data anonymity) | 16-18 |
|  | Reflexivity | N/A |
|  | Reporting guidelines used in reporting the study | 10 |
|  | Transparency in all processes | 18-19 |
| Trustworthiness | How it is achieved? Which techniques were used? | 18-19 |
|  | Credibility (e.g., prolonged engagement and member checking), transferability, dependability, conformability, and authenticity | 18-19 |
| Ethical consideration | Ethical clearance and ethical approval: document to be available per request | 19 |
|  | Participant’s confidentiality and anonymity | 19 |
|  | Informed consent and procedure of taking informed consent | 19 |
| Results | Summary and clear statement of findings | 19 |
|  | Research findings | N/A |
|  | Major and minor themes | N/A |
|  | Narration, quotes, and field notes | N/A |
|  | Diagrams, box, photographs, and video links (clear presentation of findings) | N/A |
| Discussion | Any biases (selection bias, publication bias, and heterogenity) | N/A |
|  | Interpretation of study results | N/A |
|  | Summary of major findings and comparison with the existing literature and theory | 21-22 |
|  | Alternative explanation of findings | N/A |
|  | Implication, transferability, strength, and limitation of study contribution to the field | 22-23 |
| Conclusion | Describing implications | 23 |
|  | Conclusion should come from analysis and interpretation | 23 |
| Strength and limitation | Strength and limitations | 23 |
|  | How valuable are the study results? | 23 |
| Recommendation | Recommendation for further studies and to the field | 23 |
| Funding | Source of funding and other support received during the study process | 24 |
|  | Role of funders in data collection, interpretation, and reporting funding source (financial and nonfinancial support) | 24 |
|  | Financial support for authorship or publication | 24 |
| Reference | Describe the information sources used/citations | 25 |
|  | Appendix | Appendix |
| Conflict of interest | Potential influence on the study and how it was managed | 24 |
